# The Associations between Individual Factors, eHealth Literacy, and Health Behaviors among College Students

**DOI:** 10.3390/ijerph17062108

**Published:** 2020-03-22

**Authors:** Chiao Ling Huang, Shu-Ching Yang, Chia-Hsun Chiang

**Affiliations:** 1 Faculty of Education, Department of Educational Information Technology, East China Normal University, Shanghai 200062, China; 2 Institute of Education, National Sun Yat-Sen University, Kaohsiung 80424, Taiwan

**Keywords:** college student, exercise habits, dietary behaviors

## Abstract

Background: This study aimed to investigate the associations between individual factors, electronic health (eHealth) literacy, dietary behaviors, and exercise habits in college students, as well as the moderating effect of gender on the above target behaviors. Methods: A pen-and-paper questionnaire with a stratified sampling method was used to collect data, and at least 100 students from each stratum were determined to be used for the official sample in this study. Finally, 674 students completed the survey. Results and Conclusions: Chi-square test results demonstrated that genders had dissimilar dietary supplement use and subjective health status. Further analyses indicated females had a higher likelihood of taking dietary supplements and poorer subjective health statuses. The *t*-test results indicated that the functional eHealth literacy, dietary behaviors, and exercise habits of genders were different, and the mean scores showed that males had higher functional eHealth literacy, healthier dietary behaviors, and higher exercise involvement than females. Regression analyses showed that students who were male, took dietary supplements, placed the utmost importance on health, and had high critical eHealth literacy tended to possess healthy dietary behaviors. Students who were male and had good subjective health statuses tended to have higher exercise involvement. Specifically, the critical eHealth literacy changed dietary behaviors less effectively for women than for men, and the subjective health status changed exercise habits less effectively for women than for men. Therefore, when designing the diet and exercise intervention programs, gender-specific programs rather than generic programs should be given priority to develop.

## 1. Introduction

Unhealthy dietary behaviors and poor exercise habits can contribute to many health problems later in life [1]. The identities and health habits of young people are formed during their transitions from adolescence to young adulthood [2]. The college period is the ideal time to focus on establishing positive lifelong health behaviors [3]; however, the college period is also noted for the appearance of unhealthy habits that place students at risk for poor health outcomes [4].

Many studies have revealed that college students have imbalanced diets [5,6], eat fewer than five servings of vegetables and fruits per day [7], and have poor exercise habits [1,8]. It is worrying that college students are engaging in unhealthy dietary behaviors and exercise habits that could increase their risk of health problems later in life. Therefore, investigating which factors are associated with college students’ dietary behaviors and exercise habits is imperative.

Health literacy, i.e., the capacity to obtain, comprehend, and utilize health information [9], has been identified as an important factor in healthy dietary and exercise habits. In recent decades, the internet has been widely used to obtain health-related information. To ensure that they obtain the necessary information and that the information is correct, individuals need to be health literate but also have the ability to access information using digital technology and services [10]. Thus, researchers have begun to pay attention to the relationship between electronic health (eHealth) literacy and health behaviors.

eHealth literacy consists of three levels—functional, interactive, and critical [11]—and is defined as “the ability to seek, find, understand, and appraise health information from electronic sources and apply the knowledge gained to address or solve a health problem” [12]. Studies have demonstrated that individuals with adequate eHealth literacy tended to have balanced diets and regular physical exercise [13]. In recent years, researchers have paid attention to different levels of eHealth literacy and have shown that individuals with higher functional [14] and critical eHealth literacy [14,15,16] tended to have balanced diets and adopt healthy exercise behaviors. In addition, individuals with adequate interactive eHealth literacy tended to have balanced diets [6]. Apparently, individuals with the composite skills of eHealth literacy are more likely to adopt positive health behaviors [17].

Moreover, given that individual behavior is affected by multiple factors, individual factors (e.g., gender, perception of the importance of health, subjective health status, and dietary supplement use) are considered to be influential for dietary behaviors and exercise habits. In fact, gender has been identified as the strongest predictor of health behaviors [18]. Compared to males, females have been reported to have poor nutrition behaviors [5,6,19] and poor exercise habits [15,19]. Interestingly, the literature seems to have no general consensus regarding the direction of gender differences in dietary behaviors. Unlike the former evidences we mentioned, some studies found that females have a higher intake of fruit [20] and vegetables than males [20,21]. In addition, studies have found that individuals who place the utmost importance on health tend to adopt healthy dietary and exercise behaviors [14,15]. Individuals with good subjective health statuses also tend to engage in physical exercise on a regular basis [22,23,24] and eat fruits and vegetables [24,25]. Nevertheless, paradoxical findings also existed in related research. Individuals may view dietary supplements as conferring health advantages and compensating for an unhealthy lifestyle [26]. Some studies also found that those who take dietary supplements may have healthier dietary [27,28] and exercise behaviors [28] than those who do not take supplements. The above evidences revealed that there is indeed an association between dietary behaviors, exercise habits, and individual factors.

The effects of gender, the perception of the importance of health, subjective health status, dietary supplement use, and eHealth literacy on health behaviors have received much attention. To explain the relationships among individual factors, examination of the different levels of eHealth literacy, dietary behaviors, and exercise habits is necessary. Moreover, some studies have found that gender differences exist in the perception of the importance of healthy eating [29], subjective health status [30,31,32], dietary supplement use [28,33], and health literacy [34,35]. Thus, whether gender differences exist in the perception of the importance of health, subjective health status, dietary supplement use, and eHealth literacy becomes a research question which should not be overlooked. In particular, evidence has demonstrated that gender plays an important role in health behaviors [5,6,15,18,19]; further investigation of whether the interaction of gender and these factors (the perception of the importance of health, subjective health status, dietary supplement use, and eHealth literacy) exist in health behaviors seems to also be important. Unfortunately, few studies have fully investigated these relationships regarding college students’ dietary behaviors and exercise habits. It is not clear whether the relationships between these factors and health behaviors were mediated by gender.

To bridge the research gap in this field, this study aimed to investigate the associations between individual factors, eHealth literacy, and health behaviors (dietary behaviors and exercise habits) in a college student population and further examined the moderating effect of gender on the above target behaviors. We believe that an in-depth study on these variables can not only contribute to this field of research but also help in developing a useful health program to promote college students’ healthy dietary behaviors and exercise habits.

## 2. Materials and Methods

Setting and population: This study was conducted in Taiwan, employing the questionnaire survey with a stratified sampling method to collect data. Specifically, we recruited participants from the northern, central, and southern regions and determined that at least 100 students from each region were needed for the official sample. The questionnaires were administered in class during the regularly scheduled class times. Students took 10−15 min to complete the questionnaire and received a small gift as a reward, regardless of whether they completed the survey. Notably, given that some teachers refused to help, the total number of questionnaires issued in each region was different. Ultimately, 700 college students from eight schools participated in this research. After discarding the incomplete questionnaires, 674 valid responses were obtained. The sample comprised 388 males and 286 females, and the average age of our sample was 20.44 years (SD = 2.03). Among the participants, 354 studied in the northern region, 219 studied in the central region, and 101 studied in the southern region.

Instrument: Our instrument was composed of items on participants’ demographic information, the eHealth literacy scale, and items on dietary behaviors and exercise habits. Demographic information included age, gender, dietary supplement use (one item with dichotomous response options, i.e., user, coded as 1, or nonuser, coded as 0), perception of the importance of health (one item with a five-point Likert-type response format from not at all important, coded as 1, to very important, coded as 5), and subjective health status (one item with a five-point Likert-type response format from very poor, coded as 1, to very good, coded as 5,). The degree of importance given to health was measured by one item: “How important is health according to you?” Subjective health status was measured by one item: “How well do you currently feel with respect to your health status?”

We employed the eHealth Literacy Scale (eHLS) designed by Chiang et al. [15] to assess participants’ eHealth literacy. This scale included three levels: functional (three items), interactive (four items), and critical (five items). The functional level evaluates individuals’ reading and writing skills and their understanding of basic online health information. The interactive level evaluates the degree to which individuals’ skills and abilities can be used to extract information from various forms of social online environments. The critical level evaluates the degree to which individuals’ capacities and skills can be used to critically evaluate online health information and utilize this information to make wise health decisions. The 12-item scale was scored using a five-point Likert-type response format, ranging from “strongly disagree” (coded as 1) to “strongly agree” (coded as 5). Higher scores indicated higher eHealth literacy. In the study, functional (Cronbach’s alpha = 0.81), interactive (Cronbach’s alpha = 0.84), and critical eHealth literacy (Cronbach’s alpha = 0.81) showed good internal reliability.

The dietary behavior items were developed based on the reference standard of Taiwan Ministry of Health and Welfare [36] to evaluate individuals’ nutrient intake and food choices (five items). The items were scored using a five-point Likert-type response format, ranging from “never” (coded as 1) to “always” (coded as 5). The total score range was between 5 and 25. Higher scores revealed greater alignment with the recommendations of Taiwan Ministry of Health and Welfare. The exercise habits items were developed based on a thorough review of the literature [16,37,38] to evaluate individuals’ exercise involvement (three items). To calculate the results, the formula for exercise involvement was exercise participation = motion frequency × (average intensity + exercise duration). The score range was between 1 and 6 for motion frequency, was between 1 and 5 for exercise duration, and was between 0.1 and 1 for average intensity. Thus, the total score ranged from 1.1 to 36. Higher scores indicated a higher extent of exercise involvement. For details regarding the eHealth literacy scale, dietary behavior, and exercise habits, please refer to Appendix A.

Procedure: We used a pen-and-paper questionnaire to collect data, and the questionnaire was distributed from April 2019 to May 2019. It took participants 10−15 min to complete and all procedures followed the code of research ethics.

Statistical methods: We used descriptive statistical analysis, chi-square test, independent *t*-test, and hierarchical regression analysis to address our data. Descriptive statistical analysis was performed to obtain a preliminary understanding of the respondents, such as their demographic characteristics and their health-related conditions, attitudes, behaviors, and literacy. Additionally, we examined whether gender differences existed in the above characteristics by employing the chi-square test and *t*-test. Two regression models were established to clarify the predictive effects of individual factors (e.g., gender, dietary supplement use, subjective health status, and perception of the importance of health) and eHealth literacy on dietary behaviors and exercise habits. Notably, gender was set as a moderating variable in the two models based on the literature to analyze the true associations among the criterion variables and predictor variables.

Ethical issues: This study followed the code of research ethics and conformed to the Taiwan government’s institutional review board rules for exempt review. We did not collect any relevant identifying information of the humans involved, and an anonymous design questionnaire was used in this study. The questionnaire instructions clearly informed the participants of the research purpose and their rights regarding joining or dropping out of this study. All respondents were adults, and their participation was fully based on their willingness. They could also withdraw from this study at any time without any penalty. To ensure confidentiality, all data will only be kept for 5 years and are stored in a locked cabinet that only researchers can access.

## 3. Results

### 3.1. Descriptive Statistics and Gender Differences in eHealth Literacy Dietary, Dietary Behaviors, Exercise Habits, Subjective Health Status, Perception of the Importance of Health and Supplements Use

Of the 674 participants, 56.5% reported that they took dietary supplements in their daily lives, 43.3% reported being in good or very good subjective health statuses, and 48.2% considered health to be important or very important. The descriptive statistics in Table 1 indicate that the college students had medium to high levels of eHealth literacy, but they had poor dietary behaviors and lower extent of exercise involvement.

In terms of gender differences, chi-square test analysis suggested that students with different genders have dissimilar dietary supplement use (*x*^2^ = 8.41, *p* = 0.005), and the paired comparison revealed that females tended to take dietary supplements. Since we were particularly concerned about the gender difference on subjective health status and perception of the importance of health, we applied chi-square test here rather than *t*-test to inspect whether the frequency of options is distributed identically across different genders. Result showed that students with different gender had similar perception of the importance of health (*x*^2^ = 5.66, *p* = 0.226), but dissimilar subjective health status (*x*^2^ = 34.17, *p* < 0.001). The paired comparison revealed that females tended to self-evaluate with bad or neutral health statuses, whereas males tended to self-evaluate with good or very good health statuses. Additionally, the *t*-test results showed that the functional eHealth literacy, dietary behaviors, and exercise habits of genders were different, and the mean scores showed that males had higher functional eHealth literacy, healthier dietary behaviors, and higher exercise involvement than females.

### 3.2. Regression Analysis of the Predictive Power of the Research Variables on Dietary Behavior and Exercise Habits

Before conducting the regression, we computed the values of tolerance and variance inflation factor (VIF) to ensure that no multicollinearity existed in our variables and that the values met the requirements (tolerance from 0.49 to 0.98, VIF from 1.02 to 2.03 for the first model; tolerance from 0.49 to 0.98, VIF from 1.03 to 2.06 for the second model).

The results of the two regression models are presented in Table 2 and Table 3. The analyses indicated that individual factors and the three-level eHealth literacy could jointly predict dietary behaviors and exercise habits with an overall explanatory power of 13% (*F* = 8.91, *p* < 0.001) and 16% (*F* = 10.86, *p* < 0.001), respectively.

**Figure 1:**
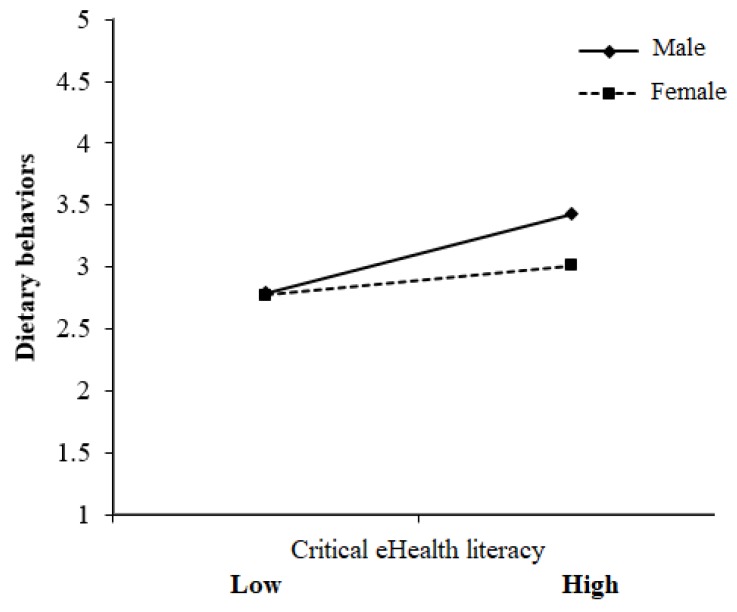
Influence of gender on critical eHealth literacy and dietary behaviors.

In the first regression model, gender (β = –0.11), dietary supplement use (β = 0.12), perception of the importance of health (β = 0.13), and critical eHealth literacy (β = 0.22) were better predictors than the other variables. Specifically, females ate fewer fruits, vegetables, and other healthy foods than with their male counterparts. In addition, those who took dietary supplements in their daily lives, paid more attention to health, and had higher critical eHealth literacy had relatively consistent healthy dietary behavior. Interestingly, an interaction existed in the relationship between critical eHealth literacy and dietary behaviors. Figure 1 shows that those who had relatively higher critical eHealth literacy had a healthier dietary behavior than those who had relatively lower critical eHealth literacy, but this relationship was moderated by gender. This finding indicates that when the critical eHealth literacy of both genders was the same, females were more likely to have poorer healthy dietary behavior than males.

**Figure 2:**
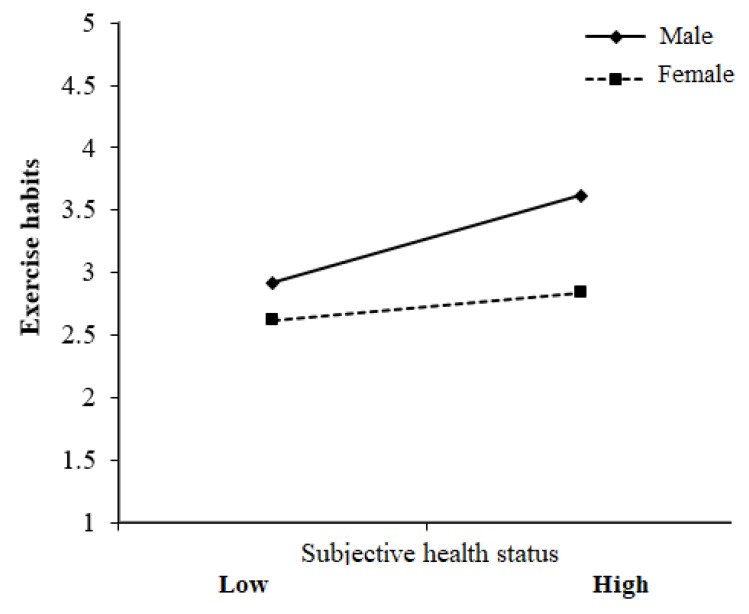
Influence of gender on subjective health status and exercise habits.

The second regression model showed that gender (β = −0.27) and subjective health status (β = 0.23) were better predictors than the other variables. In other words, females engaged in less exercise than males. In addition, those who had relatively good subjective health statuses had better exercise habits than those who had relatively poor subjective health statuses, but this relationship was moderated by gender. When the subjective health status of both genders was the same, female students had poorer exercise habits (see Figure 2).

## 4. Discussion

Our study showed that male students had better subjective health statuses than female students, which is consistent with previous studies that showed that females had poorer self-reported health than males [30,31,32]. Researchers have indicated that physical activity, the perception of health complaints, and views of well-being and quality of life may contribute to gender differences in self-reported health [31]. Many studies have revealed that female college students have lower physical activity [15,19,31], poor quality of life, and more health complaints [31] than males; therefore, the results obtained in this study are not surprising.

This study revealed that female students had a higher likelihood of dietary supplement use than male students, similar to previous studies demonstrating that females tended to use dietary supplements more than males did [28,33]. One study showed that individuals might consider dietary supplements as conferring health advantages and compensating for unhealthy lifestyles [26]. A subsequent regression analysis revealed that female students had unhealthier dietary behaviors and poorer exercise habits than male students. Thus, it is inferred that female students may use dietary supplements to try and compensate for their unhealthy lifestyles, and they are therefore more likely to take them. Beauty enhancement is one of the main motives for dietary supplement use in Taiwan [39]. Hence, female students tend to take these supplements for improvement of their looks and beautification. Future studies can further examine the reasons for their dietary supplement intake.

Moreover, male students had higher functional eHealth literacy than female students, supporting previous studies [34,35]. Functional literacy is closely related to reading comprehension and numeracy skills [40], and empirical evidence has suggested that although both genders have similar reading comprehension skills [41], males have better numeracy skills than females [41,42]. Males’ better numeracy skills may explain why male students had higher functional eHealth literacy than female students.

Notably, this study found no significant gender differences in interactive and critical eHealth literacy. Previous studies have shown that males had higher health literacy than females [34,35]. However, these studies did not further analyze whether gender differences exist in three levels of health literacy. Ample evidences indicate that individuals with higher levels of education had higher health literacy [34,35], and all our participants were college students. We inferred that education reduces the differences of eHealth literacy possessed by sexes. That is, education promotes the ability and skills of individuals to read and write (functional literacy), derive meaning from social multimedia environment (interactive literacy), and evaluate or judge the health information (critical literacy). Nevertheless, the actual effect of education to enhance three-level literacy on different genders still needs further inspection. Future studies can scrutinize whether the education improves certain eHealth literacy parameters effectively for students with specific gender.

This study revealed that the perception of the importance of health can significantly predict dietary behaviors. Previous studies have indicated that individuals who place the utmost importance on health tend to adopt healthy dietary behaviors [14,15]. Pender’s health promotion model argued that the awareness of the importance of health prompts individuals to adopt health-promoting lifestyle behaviors [22]. The perceived importance of health prompt individuals to pay attention to and maintain their health [15]; thus, the finding is reasonable.

Moreover, we found that college students who took dietary supplements were more likely to have healthy dietary behaviors. Interestingly, previous studies presented inconsistent results regarding the associations between dietary supplement use and dietary behaviors. While some people who take dietary supplements may employ healthy dietary behaviors [27,28], others may view dietary supplements as conferring health advantages and compensating for an unhealthy diet [26]. Dietary supplements are usually accompanied by advertisements and media messages about the need to “optimize” nutrition and to promote health [33]. However, excessive intake of these products may harm our bodies. For example, excessive levels of vitamin D can cause nausea and vomiting [43]. To avoid nutrient excess, individuals who take dietary supplements need to create new diet plans that divide nutrition among their food and dietary supplements. This finding alerts us to the fact that unnecessary dietary supplement use by students with healthy dietary behaviors is an issue that deserves our attention.

As expected, critical eHealth literacy was positively correlated with dietary behaviors. However, functional and interactive eHealth literacy were not correlated with dietary behaviors, which is not consistent with previous studies that showed that individuals with higher functional [14] and interactive eHealth literacy [6] tended to have balanced diets. These results indicate that the importance of critical eHealth literacy in college students’ dietary behaviors is higher than that of functional and interactive eHealth literacy for our participants. In Taiwan, one can quickly and conveniently acquire knowledge on dietary and nutritional guidelines through the Internet, news, and radio broadcasting. Critical eHealth literacy is the highest level of health literacy and refers to the capacities necessary to critically analyze online health information and to utilize this information to make appropriate health decisions [14,15]; thus, higher critical eHealth literacy may support students in adopting healthy dietary behaviors.

Notably, this study showed that gender was a significant predictor of dietary behaviors and moderated the relationship between critical eHealth literacy and dietary behaviors. Researchers argued that individuals with higher critical eHealth literacy could use health information to make good decisions about health and thereby adopt more balanced diets [14,15,16]. However, the impact of critical eHealth on dietary behaviors may vary between sexes according to our finding. When the critical eHealth literacy of both genders was the same, females were more likely to have poorer healthy dietary behavior than males. Previous studies also demonstrated that Taiwanese college girls have insufficient intake of vegetables [44] and an imbalanced diet [5,6]. The social norm that thin equals beautiful may play a major role in the lives of young females and girls, which may affect their perceptions of beauty and body image [44]. Therefore, those who equate being skinny with being beautiful tend to experience regret after eating high-calorie foods [45], and social pressure leads females to be inclined to restrict their food intake [46]. In general, girls report desires to change their bodies and weight concerns more frequently than boys [23]. Therefore, gender differences in dietary choices may partly lead to women’s greater weight-control activities [29]. Further studies need to verify the eating attitudes and habits of females with higher critical eHealth literacy, specifically, to examine how body image and critical eHealth literacy mold their perceptions as well as how body satisfaction mediates the relationship between health attitudes and eating behaviors through their critical eHealth literacy.

Surprisingly, the three levels of eHealth literacy and the perception of the importance of health were not correlated with exercise habits. These findings suggest that improving college students’ eHealth literacy and perception of the importance of health may not be sufficient to promote good exercise behaviors.

The result that subjective health status was a good predictor of individual exercise habits is consistent with previous literature [22,23,24]. According to social cognitive theory [47], an individual's behaviors are related to personal characteristics. Individuals with good subjective health statuses tend to have better physical performance, more energy and more adaptability in sports, and healthy exercise habits. Additionally, employing good exercise behaviors may lead individuals to feel healthier. Therefore, good subjective health status was correlated with healthy exercise habits.

Finally, gender not only significantly predicted individual exercise habits but also moderated the relationship between subjective health status and exercise habits. This result indicates that subjective health status changed the exercise habits of females less effectively than those of males. Dyremyhr et al. [23] showed that although there were gender differences in weight dissatisfaction, weight dissatisfaction was not related to increased time spent on physical activities. Previous studies have shown that common exercise barriers for females include lack of motivation [48,49] and disinterest in exercise [49]. Therefore, regardless of subjective health status, further studies are needed to help females improve their motivation and interest in exercise and thereby their engagement in healthy exercise habits.

## 5. Conclusions

To our knowledge, this is the first study that aimed to investigate the associations between individual factors, eHealth literacy, and health behaviors among college students and further examined the moderating effect of gender on the above target behaviors, providing a crucial starting point and insight into this research field.

This study showed that male students had better subjective health statuses and higher functional eHealth literacy than female students. By comparison, female students had a higher likelihood of dietary supplement use than male students. Furthermore, students who were male, placed the utmost importance on health, and had high critical eHealth literacy tended to adopt healthy dietary behaviors. Students who were male and had good subjective health statuses were also more likely to have higher exercise involvement. Notably, gender moderated the relationship between critical eHealth literacy and dietary behaviors and the relationship between subjective health status and exercise habits.

The findings of this research have practical implications for health education practice. Health educators needed to help college students, especially female students, place the utmost importance on health, critically evaluate health information and make healthy decisions, and thereby improve their dietary behaviors. For example, one study showed that strategies used to promote critical health literacy comprise participatory and informal learning, independent and supported assessment of a problem, appraisal of information, familiarization with health services, and social support [50], and all these are good ways for reference.

This study also showed that females were less likely to have healthy dietary behaviors and exercise habits than males. Notably, since gender moderated the relationship between critical eHealth literacy and dietary behaviors and the relationship between subjective health status and exercise habits. It should be taken into consideration when designing diet and exercise intervention programs for college students. For example, gender-specific programs rather than generic programs should be developed.

However, this study had some limitations. For example, the small sample size and the self-reported scores of dietary behaviors and exercise habits are indeed issues. Future studies should increase the sample size and develop instruments with a validated scoring system. Taking into account that students may still live with their parents, and their dietary behaviors and exercise habits may strongly be influenced by their family even before and after the college period. Future studies should also consider distinguishing the difference between students with different living arrangements. In addition, the predictive powers of dietary behavior and exercise habits were low, implying that the factors related to health behaviors are complex and interdependent. Thus, we recommend that future studies could further seek and examine the potential determinants.

## Figures and Tables

**Table 1:** Descriptive statistics and *t*-test results for our sample.

	**Whole population**	**Male**	**Female**	***t***	***p***		***d***
	*M* (SD)						
**eHealth literacy (three levels)**							
functional	3.94 (0.77)	4.04 (0.77)	3.81 (0.76)	3.84	<0.001		0.30
interactive	3.66 (0.74)	3.65 (0.75)	3.67 (0.72)	−0.254	0.799		−0.03
critical	3.78 (0.79)	3.77 (0.83)	3.79 (0.72)	−0.201	0.841		−0.03
**Dietary behaviors**	2.94 (0.65)	3.01 (0.64)	2.85 (0.65)	3.35	0.001		0.25
**Exercise habits**	9.16 (6.77)	11.02 (7.33)	6.65 (4.92)	9.26	<0.001		0.70
	**Whole population**	**Male**	**Female**	***x*^2^**		***p***	
	N (%)						
**Subjective health status (five status)**				34.17		<0.001	
very good	44 (6.50)	36 (9.30)	8 (2.80)				
good	248 (36.80)	164 (42.30)	84 (29.40)				
neutral	296 (43.90)	154 (39.70)	142 (49.70)				
bad	79 (11.70)	30 (7.70)	49 (17.10)				
very bad	7 (1.00)	4 (1.00)	3 (1.00)				
**Perception of the importance of health (five conditions)**				5.66		0.226	
very important	59 (8.80)	41 (10.60)	18 (6.30)				
important	265 (39.40)	151 (38.90)	114 (40.00)				
neutral	312 (46.40)	178 (45.90)	134 (47.00)				
not important	31 (4.60)	14 (3.60)	17 (6.00)				
not at all important	6 (0.90)	4 (1.00)	2 (0.07)				
**Dietary supplement use (two conditions)**				8.41		0.005	
user	380 (56.50)	201 (51.80)	179 (63.00)				
nonuser	292 (43.50)	187 (48.20)	105 (37.00)				

**Table 2:** Hierarchical regression analysis of dietary behaviors.

	Dietary Behaviors (*n* = 674)			
	B	SE	Beta	*p*
Gender (0 = Male, 1 = Female)	−0.07	0.03	−0.11	0.003
Dietary supplement use (0 = Nonuser, 1 = User)	0.08	0.02	0.12	0.001
Subjective health status	0.05	0.03	0.08	0.051
Perception of the importance of health	0.08	0.03	0.13	0.004
Functional eHealth literacy	0.02	0.03	0.04	0.357
Interactive eHealth literacy	0.00	0.03	0.00	0.943
Critical eHealth literacy	0.14	0.03	0.22	<0.001
Gender × Dietary supplement use	−0.01	0.02	−0.01	0.720
Gender × Subjective health status	−0.02	0.03	−0.03	0.546
Gender × Perception of the importance of health	0.02	0.03	0.03	0.428
Gender × Functional eHealth literacy	0.02	0.03	0.03	0.407
Gender × Interactive eHealth literacy	0.06	0.03	0.09	0.090
Gender × Critical eHealth literacy	−0.07	0.03	−0.10	0.038
Adjusted *R*^2^ = 0.13, *F* = 8.91, *p* < 0.001				

**Table 3:** Hierarchical regression analysis of exercise habits.

	Exercise Habits (*n* = 674)			
	B	SE	Beta	*p*
Gender (0 = Male, 1 = Female)	−1.84	0.25	−0.27	<0.001
Dietary supplement use (0 = Nonuser, 1 = User)	−0.01	0.24	−0.00	0.967
Subjective health status	1.57	0.29	0.23	<0.001
Perception of the importance of health	0.03	0.29	0.01	0.912
Functional eHealth literacy	0.27	0.26	0.04	0.300
Interactive eHealth literacy	−0.04	0.34	−0.01	0.909
Critical eHealth literacy	0.38	0.33	0.06	0.258
Gender × Dietary supplement use	−0.09	0.25	−0.01	0.719
Gender × Subjective health status	−0.84	0.28	−0.12	0.003
Gender × Perception of the importance of health	0.47	0.29	0.07	0.104
Gender × Functional eHealth literacy	−0.05	0.26	−0.01	0.843
Gender × Interactive eHealth literacy	0.15	0.34	0.02	0.659
Gender × Critical eHealth literacy	−0.33	0.34	−0.05	0.336
Adjusted *R*^2^ = 0.16, *F* = 10.86, *p* < 0.001				

## Appendix A. Instruments Used in This Study

I. **eHealth Literacy Scale (Five-Point Likert-Type)**
  1. *I cannot understand the symbols (such as BMI, Body Mass Index) and wording about health information.*
  2. *I find the online health information difficult to understand.*
  3. *I find the mathematical formulas provided in online health information difficult to calculate. (e.g., the algorithm of calorie consumption, BMI).*
  4. *I can locate health information efficiently through search engines.*
  5. *I pay attention to and obtain new knowledge about online health information.*
  6. *I know how to get what I need from online health information.*
  7. *I understand the online health information I have obtained.*
  8. *I will think about whether the online health information applies to my situation.*
  9. *I try to find different sources to verify the credibility of health information.*
  10. *I evaluate the validity and reliability of online health information.*
  11. *I will browse various discussions and make a decision or action that is good for health.*
  12. *When I have questions or doubts about online health information, I use other channels to verify the information.*
II. **Dietary Behaviors (Five-Point Likert-Type)**
  1. *More than two bowls per day of fruit.*
  2. *More than three bowls per day of vegetables.*
  3. *More than five bowls per day of meats and protein.*
  4. *More than one bowl per day of unrefined whole grains.*
  5. *More than 1.5 glasses (360 mL) per day of milk.*
III. **Exercise Habits**
  1. *Motion frequency of a week*
      (1) none (2) one day (3) two days (4) three days (5) four days (6) five or more days
  2. *Exercise duration each time*
      (1) 0−29 min (2) 30−60 min (3) 61−90 min (4) 91−120 min (5) 121 or more min
  3. *Average intensity each time*
      (1) 10% (2) 20% (3) 30% (4) 40% (5) 50%
      (6) 60% (7) 70% (8) 80% (9) 90% (10) 100%
